# Supplementary material for: Mortality in rural coastal Kenya measured using the Kilifi Health and Demographic Surveillance System: a 16-year descriptive analysis
Source: Wellcome Open Res. 2023 Feb 17;6:327. Originally published 2021 Nov 29. [Version 2] doi: 10.12688/wellcomeopenres.17307.2 (PMC10320326; doi:10.12688/wellcomeopenres.17307.2)
Supplement: Peer review history

# Peer review: Reviewer response for version 2

**DOI:** 10.21956/wellcomeopenres.20971.r60389

**Published:** 2023-07-04

**Authors:** Alain Vandormael

**Affiliations:** 1 Heidelberg University, Im Neuenheimer Feld, Heidelberg, Germany

Introduction

1. The introduction is clear and well-written.

Methods

1. The authors write “Subsequently, the population has been continuously monitored for births, pregnancies, deaths, and migration events in re-enumeration rounds occurring approximately every 4 months, and the mapping was updated in 2017.” Is this first sentence saying that all data points (births, pregnancies, deaths) are collected from the re-enumeration rounds, or just the migration data?
2. In the next paragraph, the authors write that there are 4 points of contact, where re-enumeration rounds refer to household visits. Births and pregnancies information is collected from the maternity wards. This sentence suggests that not all data is collected from the re-enumeration rounds (see comment #1). Please fix this to make the sentence and data collection methods clearer.
3. What are the four-month re-enumeration rounds referring to specifically? The average time between household visits or the average time between data points (not just household data from a key informant but also from clinics and maternity wards). This is not clear.
4. Do field workers visit only the households every for months, or do they also go to the wards, clinics and vaccination sites every four months. This is not clear.
5. The authors note that all household members can be absent during a household visit. No data or information is given on participation rates in the surveillance program. Can more be said about this? Is the household participation rate low, moderate or good? Is there a paper from the Kilifi surveillance that documents and quantifies missed household visits and non-response rates in more detail? For example, at the Welcome Trust Surveillance site in northern Kwazulu-Natal, South Africa, there is the paper by Larmarange *et al.* ^1^ that does this.
  (I am not asking for such a study to be done.) Has a previous study been done that you can reference? The reader will observe the high rates of missed HIV test dates and household visits in South Africa Welcome Trust site. Is it similar in current surveillance site? Can the authors say something about missing data in the Methods section and how it was dealt with.

Statistical Methods

1. Overall, the authors provide a clear description and justification of the statistical methods used.
2. How was missing data dealt with in the statistical methods? The authors talk about dealing with outmigration, but not about missed household visits, etc. I expect there must be a non-negligible amount of missing data.
3. How was data from the clinics and maternity wards matched, merged, and collated with data from the household visits. What was the match rate? For example, how was participant data from medical records linked to household data records. What % of these records, identifiers could be matched? Can more be said about this data quality aspect of the study.  

Results

1. The results are clearly presented. The plots showing trends and the maps showing changes in outcomes over time are informative.
2. Minor comments: Median survival increased from 65 *years” in 2003 to 77 *years* in 2018 (pg 5).

Discussion

1. The authors write: “This pattern is consistent with mortality sex ratios before and after the rapid initiation of HIV care and antiretroviral therapy in LMICs between 2003 and 2018 with evidence showing that women have benefitted more from the expansion of HIV treatment programs than men.” It is not possible that the references given (32,33) can be used to support this claim, since they were published well before the 2003-2018 period end. Moreover, in a generalized epidemic with heterosexual transmission, we would expect men to benefit more if HIV treatment is increased among women. Indeed, recent evidence ^2^ suggests that men have been benefitting more from the increased HIV care expansion among women from 2013 to 2018.
2. The authors write: “In Kilifi, the prevalence of HIV infection among women attending antenatal at KCH ranged between 3.8–4.4% between 2005–2009 and 2.0–3.7% between 2010–2016 with a clear decline from 2010 5.” Can the authors elaborate more on this HIV prevalence trend. If women are living longer over the study period, and HIV care is being expanded among women, then we would expect HIV prevalence to increase (not decrease) since HIV-positive women on treatment are expected to live longer. For the relationship between ART coverage and HIV prevalence, see for example: Zaidi *et al.,* ^3^.
3. This paper was written in 2023. Are there studies that have been undertaken in the current surveillance area that report on ART coverage and other public health services such as voluntary medical male circumcision (other than malaria prevention scale-up). While I understand that this is a descriptive analysis or mortality trends in the surveillance area, it would be useful for the reader if the authors can hypothesize why they have seen improvements in mortality over the last 16 years. It is highly likely that the scale-up of HIV treatment and prevention strategies is an important part of this story. The authors touch on this point lightly and with respect to LMICs, but what about saying more about the potential impact of these programs specifically in the surveillance area.

Is the work clearly and accurately presented and does it cite the current literature?

Yes

If applicable, is the statistical analysis and its interpretation appropriate?

Yes

Are all the source data underlying the results available to ensure full reproducibility?

No

Is the study design appropriate and is the work technically sound?

Yes

Are the conclusions drawn adequately supported by the results?

Partly

Are sufficient details of methods and analysis provided to allow replication by others?

Yes

Reviewer Expertise:

HIV epidemiology

I confirm that I have read this submission and believe that I have an appropriate level of expertise to confirm that it is of an acceptable scientific standard.

## References

[1] : Participation dynamics in population-based longitudinal HIV surveillance in rural South Africa. *PLoS One* .2015;10(4) : 10.1371/journal.pone.0123345 e0123345 10.1371/journal.pone.0123345 25875851PMC4395370

[2] : Declines in HIV incidence among men and women in a South African population-based cohort. *Nat Commun* .2019;10(1) : 10.1038/s41467-019-13473-y 5482 10.1038/s41467-019-13473-y 31792217PMC6889466

[3] : Dramatic increase in HIV prevalence after scale-up of antiretroviral treatment. *AIDS* .2013;27(14) : 10.1097/QAD.0b013e328362e832 2301-5 10.1097/QAD.0b013e328362e832 23669155PMC4264533

# Peer review: Reviewer response for version 2

**DOI:** 10.21956/wellcomeopenres.20971.r60394

**Published:** 2023-07-04

**Authors:** Ian Cook

**Affiliations:** 1 University of Limpopo, Mankweng, Limpopo, South Africa

**General comments:**

 Well written and constructed paper - achieves a balance between detail and overview. Also, caters for a specialised reader as well as a more general readership. Notable, the number of publications which have emanated from the KDHSS. A large database with almost 300 000 residents in 2018, 700 000 individuals and nearly 4 million p-y of observation, analysed over 16 years. 

**Specific comments:**

- It might be useful (if even in an appendix) to provide a map(s) (country and county), identifying the site and important landmarks (hospitals/towns, cities/major transport routes etc) in the site area. And the location of the other Kenyan INDEPTH sites.
- The authors mention the use of INDEPTH methodology, but do not explicitly state that the KHDSS is part of the INDEPTH network of sites ( http://www.indepth-network.org/member-centres). I note there are five HDSS sites in Kenya (KHDSS included) - yet the discussion does not explicitly compare (even briefly) the results with that of other Kenyan INDEPTH sites. If there are no comparable Kenyan INDEPTH data, the authors might consider explicitly stating this.
- Sufficient figures, which are clear and easily interpretable, to highlight trends.

Is the work clearly and accurately presented and does it cite the current literature?

Yes

If applicable, is the statistical analysis and its interpretation appropriate?

I cannot comment. A qualified statistician is required.

Are all the source data underlying the results available to ensure full reproducibility?

Yes

Is the study design appropriate and is the work technically sound?

Yes

Are the conclusions drawn adequately supported by the results?

Yes

Are sufficient details of methods and analysis provided to allow replication by others?

Yes

Reviewer Expertise:

Public Health, Physical Behaviours Epidemiology

I confirm that I have read this submission and believe that I have an appropriate level of expertise to confirm that it is of an acceptable scientific standard.

# Peer review: Reviewer response for version 1

**DOI:** 10.21956/wellcomeopenres.19134.r47348

**Published:** 2022-01-14

**Authors:** Momodou Jasseh

**Affiliations:** 1 Medical Research Council Unit The Gambia at LSHTM, Fajara, The Gambia

**Mortality in rural coastal Kenya measured using the Kilifi Health and Demographic Surveillance System: a 16-year descriptive analysis**

 The authors claim the paper to be the first attempt ever to systematically characterise general mortality in the Kilifi HDSS since it was established in 2000. Being an area that has hosted a significant number of important trials and health interventions of the KEMRI-Wellcome Trust Research Programme, and with access to tertiary health care through the Kilifi County Hospital, a systematic description of levels and trends in all-cause mortality in the area is necessary and relevant; and the attempt is therefore justified.

 Whilst the paper is generally well-written, it can be enhanced further both in terms of content and presentation as indicated in the following queries.

**GENERAL QUERIES**

1. The frequent reference made to supplementary tables and figures in the paper makes reading it a rather arduous task. If tables and figures are important enough to be presented as part of the results, then they should appear in the paper accordingly. The authors should therefore decide what information to include in the paper and how they should be lucidly presented to enhance clarity and ease comprehension.
2. None of the mortality rates estimated and shown in the tables and graphs are presented with Confidence Intervals (95% CI). It is recommended that these be provided for all indicator estimates presented in tables, and as CI bars in levels/trends graphs. Not only will they add value to the results of the study but will also enable the reader to make inferences/deductions from the presented results beyond what the authors have reported, especially – for example – with respect to confirming statistically significant differences between indicators for different time periods.
3. I observe the interchangeable use of the terms “rates” and “ratios”. Since mortality indicators are conventionally estimated as rates, I will suggest that the term “rates” be used consistently in the paper.
4. Too many methods are used to characterise mortality for the first time in the Kilifi HDSS area. The purpose of the paper is mortality estimation and validation of the KHDSS data, and not comparison of methods using the same dataset. Therefore, it may be worthwhile to adopt the conventional methods of mortality estimation to effectively describe the characteristics of the region’s mortality experience for the stated period; as well as facilitate easy comparison with estimates from other data sources, and for other regions in Kenya. For instance, comparisons of life expectancy estimates from the Life Table method with similar estimates from census or DHS sources provides more analytical value in assessing the quality of the KHDSS data than comparing them with Kaplan-Meier-derived mean life expectancies from the same dataset. The Life Table method also provides the opportunity to cross-check and validate derived age-specific mortality rates.

**SPECIFIC QUERIES**

1. *Page 4, left column, fifth para*: Remove the word “survival” from the sub-title. It can be used interchangeably with “mortality” and the choropleth maps refer to mortality rates over time.
2. *Page 4, left column, fifth para*: Provide a justification for applying the direct standardization of the populations against the 2011 KHDSS age structure. If the idea is to use the mid-period age structure for this purpose, then that of 1 ^st^ January 2013 should be used instead.
3. *Pages 5 & 6*: Consider merging Tables 1 and 2 to provide comprehensive information on births, deaths, PYO and corresponding mortality rates (with 95% CI) by age-group and period. The childhood mortality estimates (i.e. neonatal, post-neonatal, infant [<1 yr]), child [1-4 yrs] and under-5) can be expressed in both per 1,000 PYO and per 1,000 live births where applicable. The authors are urged to reconsider the method used in estimating the childhood mortality rates presented in Table S2. These rates should be equivalent to the probabilities of dying within the respective age brackets, and can be estimated more accurately using the Lexis Diagram method, for instance. The under-5 mortality rate should not be the sum of the infant mortality (<1) and child mortality (1-4), but rather the complement of the product of the probability of surviving the first year of life and the probability of surviving to exact age 5 thereafter. The authors should therefore review their method of analysis to provide accurate measures of childhood mortality indicators. In fact, the mortality rate for children aged 1-4 years cannot be expressed in “per 1,000 live births”, because it refers to a population defined by having already survived the first year of life.
4. *Page 5, left column, last para*: The statement that a seasonal pattern appears only in children aged 1-4 years in the period 2003-2006 is speculative. Whether it is real or a data artefact can be ascertained by adopting more robust statistical analysis. An inclusion of 95% CI bars in Figure 3 would have enabled the reader to make an inference to confirm or dismiss such a seasonal mortality pattern claim.
5. *Page 10, right column, second para*: The explanation linking oestrogen levels and female life expectancy is beyond the scope of the paper and should be removed.
6. Whilst sufficient comparison is done between KHDSS childhood mortality indicators with estimates from other sources and regions, not much similar comparison is attempted for adult mortality.

Is the work clearly and accurately presented and does it cite the current literature?

Partly

If applicable, is the statistical analysis and its interpretation appropriate?

Partly

Are all the source data underlying the results available to ensure full reproducibility?

Yes

Is the study design appropriate and is the work technically sound?

Partly

Are the conclusions drawn adequately supported by the results?

Partly

Are sufficient details of methods and analysis provided to allow replication by others?

Partly

Reviewer Expertise:

Demography

I confirm that I have read this submission and believe that I have an appropriate level of expertise to confirm that it is of an acceptable scientific standard, however I have significant reservations, as outlined above.

# Response

**Published:** 2023-01-20

**Authors:** Mark Otiende

**Affiliations:** KEMRI Wellcome Trust Reseacrh Program, Kenya

**Response to the reviewer**

 We thank the reviewer for his interest in our paper and for taking the time to review it thoroughly. We have given point-by-point responses in italics.

**Mortality in rural coastal Kenya measured using the Kilifi Health and Demographic Surveillance System: a 16-year descriptive analysis**

 The authors claim the paper to be the first attempt ever to systematically characterise general mortality in the Kilifi HDSS since it was established in 2000. Being an area that has hosted a significant number of important trials and health interventions of the KEMRI-Wellcome Trust Research Programme, and with access to tertiary health care through the Kilifi County Hospital, a systematic description of levels and trends in all-cause mortality in the area is necessary and relevant; and the attempt is therefore justified.

 Whilst the paper is generally well-written, it can be enhanced further both in terms of content and presentation as indicated in the following queries.

**GENERAL QUERIES**

1. The frequent reference made to supplementary tables and figures in the paper makes reading it a rather arduous task. If tables and figures are important enough to be presented as part of the results, then they should appear in the paper accordingly. The authors should therefore decide what information to include in the paper and how they should be lucidly presented to enhance clarity and ease comprehension.
*As mentioned in the review, Kilifi HDSS is the setting for a large number of studies of observational epidemiology and public health interventions and scientists reading these studies may wish to interrogate the background mortality experience of the population from a variety of different perspectives. This is what has motivated our analyses – this is expressed in the introduction. We divided the results between the main paper and a supplement to produce a main paper that was accessible to the general reader and simultaneously provide a compendium of tabulation sufficiently comprehensive to satisfy the more detailed analyst. We have decided what information we wish to emphasise and provided signposts to the supplementary data merely to inform the more curious reader. *
2. None of the mortality rates estimated and shown in the tables and graphs are presented with Confidence Intervals (95% CI). It is recommended that these be provided for all indicator estimates presented in tables, and as CI bars in levels/trends graphs. Not only will they add value to the results of the study but will also enable the reader to make inferences/deductions from the presented results beyond what the authors have reported, especially – for example – with respect to confirming statistically significant differences between indicators for different time periods.
*In our analysis, the Kilifi HDSS is both the population of interest and the sample population. Since we are not inferring to a larger population, confidence intervals for our mortality rates estimates are unnecessary. However, we agree with the reviewer that it is important to rule out month-to-month fluctuations (e.g. due to conceptions, temperature, rainfall, crop yields, road access to hospital, vaccination coverage, etc) when comparing between time periods. To rule out random fluctuations (Table 2 and Figure 3a), we have fitted a negative binomial model with yearly rates as the outcome (yearly rates eliminate the issue of seasonality) and period as the explanatory variable. For all age groups under 55 years, we observed significantly lower mortality rates in the latter periods compared to the first period (2003-2006). We have added a supplementary table (Table S2) showing these comparisons for all age groups.*
3. I observe the interchangeable use of the terms “rates” and “ratios”. Since mortality indicators are conventionally estimated as rates, I will suggest that the term “rates” be used consistently in the paper.
*We have emphasised the calculation of rates because we have a sophisticated dataset with events linked to person years at risk. Much demographic data does not have this linkage and many of the estimates of mortality are calculated as the ratio of the number of deaths in any one age group in one place in one year to the number of live births in the same place in the same year. These ratios are used as an approximation of the cumulative mortality incidence but they have a number of shortcomings including the fact that where the birth rate fluctuates annually, they misrepresent the denominator for any group beyond infancy, and the fact that they calculate the mortality based on the starting population not the person years at risk. From this perspective they are risks (not rates) but the lack of correspondence between the numerator and denominator negates this description and so we, and others* *^1,2^* *, have referred to these as ratios. As much demographic data is captured with this method we have calculated the ratios in Kilifi for wider comparison. We have not used the terms interchangeably and in fact we defined their separate and distinct use in the statistical methods (Age-sex mortality profile); the first paragraph defines the calculation of rates, the second the calculation of (and justification for) ratios. *
4. Too many methods are used to characterise mortality for the first time in the Kilifi HDSS area. The purpose of the paper is mortality estimation and validation of the KHDSS data, and not comparison of methods using the same dataset. Therefore, it may be worthwhile to adopt the conventional methods of mortality estimation to effectively describe the characteristics of the region’s mortality experience for the stated period; as well as facilitate easy comparison with estimates from other data sources, and for other regions in Kenya. For instance, comparisons of life expectancy estimates from the Life Table method with similar estimates from census or DHS sources provides more analytical value in assessing the quality of the KHDSS data than comparing them with Kaplan-Meier-derived mean life expectancies from the same dataset. The Life Table method also provides the opportunity to cross-check and validate derived age-specific mortality rates.
*In writing this paper our purpose was two-fold. We wanted to provide a digestible description of the mortality patterns and trends for those who have read the many epidemiological studies and intervention trials that have taken place in Kilifi and we wanted to provide the comprehensive demographic tables to allow demographers to compare our data with other published estimates. As many of the prior studies in Kilifi have been epidemiological, the readers are familiar with survival analyses and the visual efficiency with which a Kaplan Meier curve can convey information on (instantaneous) mortality rates by age. Conventional analyses using life tables, by contrast, provide tabulated discrete data in many strata that are less easy for the general reader to assimilate rapidly but are necessary for the analysis of standard demographic outputs. In line with our purpose to make the paper digestible, we have emphasised the presentation of survival curves in the paper and in line with our purpose to be comprehensive we have included a full set of life tables in the appendix. We accept that, to one audience segment, the survival analyses may seem superfluous, and to the other the life tables may seem inaccessible but we aim to reach both and we invite our readers to take note, that the two approaches provide slightly different estimates of life expectancy – and we discuss this briefly.*

**SPECIFIC QUERIES**

1. *Page 4, left column, fifth para*: Remove the word “survival” from the sub-title. It can be used interchangeably with “mortality” and the choropleth maps refer to mortality rates over time.
*Following directly from the response above – we have tried to signpost which analytic approach is being used at each point. The sub-header ‘Survival’ precedes a paragraph explaining the survival analysis of the mortality data and a reference to six Kaplan Meier survival curves. Later, under the heading Geographic heterogeneity in survival and mortality we signpost the variation in survival (from the survival analyses which we detail in the supplement) and then present here the location-specific mortality rates in the choropleth maps. Our judgment is that, whilst survival curves describe mortality by age efficiently, mortality rates provide a more efficient metric to understand the variation of mortality cartographically.*
2. *Page 4, left column, fifth para: * Provide a justification for applying the direct standardization of the populations against the 2011 KHDSS age structure. If the idea is to use the mid-period age structure for this purpose, then that of 1st January 2013 should be used instead.
*We stand by our calculation – for a period beginning 1st January 2003 and ending 31st December 2018, a mid-point estimate of 1st January 2011 is perfectly reasonable (it could be argued that 31st December 2010 may be more accurate – in days…) The purpose is to find a reasonable reference population for a standardisation exercise – it is not necessary to fix it to the mid-study point, though this is what we did.*
3. *Pages 5 & 6: * Consider merging Tables 1 and 2 to provide comprehensive information on births, deaths, PYO and corresponding mortality rates (with 95% CI) by age-group and period. The childhood mortality estimates (i.e. neonatal, post-neonatal, infant [<1 yr]), child [1-4 yrs] and under-5) can be expressed in both per 1,000 PYO and per 1,000 live births where applicable. The authors are urged to reconsider the method used in estimating the childhood mortality rates presented in Table S2. These rates should be equivalent to the probabilities of dying within the respective age brackets, and can be estimated more accurately using the Lexis Diagram method, for instance. The under-5 mortality rate should not be the sum of the infant mortality (<1) and child mortality (1-4), but rather the complement of the product of the probability of surviving the first year of life and the probability of surviving to exact age 5 thereafter. The authors should therefore review their method of analysis to provide accurate measures of childhood mortality indicators. In fact, the mortality rate for children aged 1-4 years cannot be expressed in “per 1,000 live births”, because it refers to a population defined by having already survived the first year of life.
*We have extended Table 2 to include age and period specific numbers of deaths and pyo in addition to rates. In our selection of results we have emphasised mortality rates (over ratios) for the reasons outlined in general query #3 above and have only presented the infant mortality ratio, under 5 mortality ratio etc for the sake of comparison with other demographic sources, and only in the supplement. We agree with the reviewer that these ratios provide a metric which has no basis in reality (‘the mortality rate in children aged 1-4 years cannot be expressed…’) but this analytic approach is, nonetheless, in widespread use and is frequently taught (see for example the definition of ‘Post-neonatal mortality rate’ at* *https://www.cdc.gov/csels/dsepd/ss1978/lesson3/section3.html* *). For exact probabilities of dying from one age point to the next, we refer our readers to the Life Tables in the supplement.*
4. *Page 5, left column, last para*: The statement that a seasonal pattern appears only in children aged 1-4 years in the period 2003-2006 is speculative. Whether it is real or a data artefact can be ascertained by adopting more robust statistical analysis. An inclusion of 95% CI bars in Figure 3 would have enabled the reader to make an inference to confirm or dismiss such a seasonal mortality pattern claim.
*In addition to the visual interpretation of the data (a seasonal pattern appearing only in in children aged 1-4 years), results from a negative binomial regression model confirm a significant interaction between season and period in children aged 1-4 years only. We have reported the same in the in the methods, results and supplement.*
5. *Page 10, right column, second para: * The explanation linking oestrogen levels and female life expectancy is beyond the scope of the paper and should be removed.
*We have removed it.*
6. Whilst sufficient comparison is done between KHDSS childhood mortality indicators with estimates from other sources and regions, not much similar comparison is attempted for adult mortality.
*There is a lot less data on adult mortality compared to child mortality which has made it difficult to interpret these estimates. We have made this point in the discussion (paragraph 2) and also cited adult mortality estimates derived from DHS sibling survival data.*

# Peer review: Reviewer response for version 1

**DOI:** 10.21956/wellcomeopenres.19134.r47346

**Published:** 2021-12-16

**Authors:** Stephen Gordon

**Affiliations:** 1 Department of Clinical Sciences, Liverpool School of Tropical Medicine, Liverpool, UK; 2 Malawi Liverpool Wellcome Programme, Blantyre, Malawi

General comments: Elegant, detailed and clear account of a large well conducted study. Many high quality publications have emerged from the Kilifi DHSS which has become a model for many others.

 Specific comments

 Title and Abstract are clear. The abstract would be improved by including the size of the dataset.

 Introduction: Clear, but could be improved by a little bit of geography to explain the terrain, who the people are, a bit more colour!

 Methods: Clear, and a good explanation of how to deal with in- and out-migration.

 Results: 22207 deaths and 3.9m pyo is a very big study. It is good to see such a precise data set to support other data based on less solid observations. I note the lack of seasonality, which is different from that published from Gambia. Would you like to comment? Geographical variation is found everywhere (e.g. in UK) and often relates to poverty. Could you look at poverty, clean water or distance to health facilities in your analysis?

 Discussion: Nice discussion of HIV, vaccination and gender. I'm curious about the north/south divide that has emerged across the creek with the north showing more improvement. Can you comment? Good comparison with other data sources and explanation of differences.

 Figures: elegant and helpful.

Is the work clearly and accurately presented and does it cite the current literature?

Yes

If applicable, is the statistical analysis and its interpretation appropriate?

I cannot comment. A qualified statistician is required.

Are all the source data underlying the results available to ensure full reproducibility?

Yes

Is the study design appropriate and is the work technically sound?

Yes

Are the conclusions drawn adequately supported by the results?

Yes

Are sufficient details of methods and analysis provided to allow replication by others?

Yes

Reviewer Expertise:

Respiratory Medicine and general internal medicine, work in Low Income Countries.

I confirm that I have read this submission and believe that I have an appropriate level of expertise to confirm that it is of an acceptable scientific standard.
